# Comparison of pharyngeal and invasive isolates of *Streptococcus pyogenes* by whole-genome sequencing in Toronto, Canada

**DOI:** 10.1128/spectrum.02141-24

**Published:** 2025-02-13

**Authors:** Joseph J. Zeppa, Ellen G. Avery, Patryk Aftanas, Erin Choi, Simone Uleckas, Prachi Patel, Nicholas Waglechner, Hubert Jimenez, Christie Vermeiren, Kevin Katz, Xena X. Li, Finlay Maguire, Robert Kozak

**Affiliations:** 1 Department of Laboratory Medicine & Pathobiology, University of Toronto 7938 , Toronto, Ontario, Canada; 2 Shared Hospital Laboratory, Toronto, Ontario, Canada; 3 Laboratory Medicine and Molecular Diagnostics, Sunnybrook Health Sciences Centre 71545 , Toronto, Ontario, Canada; 4 Department of Community Health & Epidemiology, Dalhousie University, Halifax, Nova Scotia, Canada; 5 Faculty of Computer Science, Dalhousie University, Halifax, Nova Scotia, Canada; University of Rome, P.le Aldo Moro, Rome, Italy

**Keywords:** pathogenesis, *Streptococcus pyogenes*, whole-genome sequencing, virulence factors, non-invasive isolates

## Abstract

Invasive Group A streptococcal (iGAS) infections are rising in Canada and wordwide. The 2022–2023 Ontario iGAS season was among the highest recorded, a trend continuing in 2023–2024. We sequenced 38 invasive (blood) and 117 non-invasive (pharyngeal) *Streptococcus pyogenes* clinical isolates from Toronto (January–May 2023) to compare between the two cohorts and against published sequences to determine if any genomic changes accounted for the trend. Results demonstrated limited clustering with one small totally invasive cluster (*emm*49) with both invasive and non-invasive isolates represented across a diverse set of lineages. Non-invasive isolates were predominantly *emm12* (70.1%), whereas invasive isolates included emm*12* (26.32%), *emm49* (23.68%), and *emm1* (13.16%) with most *emm1* strains containing the 27 SNPs that define the hypervirulent M1_UK_ clone (58.33%). Although there were no differences in the presence of overall virulence factors/adhesin genes between cohorts, there were statistically more superantigen and DNase genes in non-invasive isolates and a rare phage gene was significantly associated with invasiveness across three *emm*-types. The prevalence of individual virulence factor/adhesin genes also differed between our cohorts, including a higher likelihood of *speA*, *enn*, *mrp*, *ideS/Mac*, *fbaA*, and *fbaB* in invasive isolates. There were also no significant differences across the 11 antimicrobial resistance genes identified. Finally, pharyngeal isolates had larger hydrolysis and hemolysis zones, and *covS* deletions were observed in only seven invasive strains. Despite there being no genetic signature that differentiated our isolates, we observed several features that were predominant in invasive strains which provides further insights into the factors that contribute to GAS invasiveness.

**IMPORTANCE:**

Increasing rates of invasive Group A streptococcal (iGAS) infections are being seen both in Canada and worldwide, which is leading to a greater disease burden caused by this pathogen. Leveraging whole-genome sequencing gives us an opportunity to better understand the underlying genetic mechanisms of streptococcal disease. By utilizing this technique, we shed light on the circulating invasive and non-invasive strains of *Streptococcus pyogenes* in the largest urban area in Canada from January to May 2023. GAS strains causing non-invasive disease were found to have a higher abundance of superantigen and DNase genes, whereas invasive isolates were more likely to contain M-like protein genes, the superantigen *speA*, the protease *ideS/Mac,* and/or the fibronectin-binding proteins *fbaA* and *fbaB*. This work provides valuable insights into iGAS disease which will help with surveillance, epidemiology as well as developing treatment and preventative modalities to help curb the disease burden caused by this globally important pathogen.

## INTRODUCTION

Group A streptococcus (GAS; *Streptococcus pyogenes*) is a human-specific pathogen of global importance, capable of causing numerous disease manifestations from superficial infections such as pharyngitis to more severe invasive diseases like necrotizing fasciitis and toxic shock syndrome. The ability of this organism to cause such a wide variety of infections is attributed to its diverse set of virulence factors, and its ability to colonize almost every tissue in the body (1, 2). Despite being universally susceptible to penicillin-class antibiotics, this organism is still responsible for over 500,000 deaths each year worldwide (3).

Recently, there has been an increase in the number of invasive GAS (iGAS) cases worldwide (4–6). iGAS is defined as the isolation of the organism or its nucleic acid from a normally sterile site (i.e., the blood). In 2016, a rise of iGAS cases in the United Kingdom led to the discovery of a more virulent strain that was shown to upregulate key virulence factors, including the superantigen SpeA (7). Using the gold standard typing scheme for GAS, *emm-*typing, which involves sequencing the hypervariable N-terminus of the key surface virulence factor M protein, this strain was found to be a unique offshoot of the *emm1*-subtype and was termed M1_UK_. This clone has been found in numerous countries (8), including Canada (9), and is associated with this increase in invasive disease. In Ontario specifically, the 2022–2023, iGAS season (October 2022 to September 2023) had the highest number of monthly cases in all but 2 months compared to the five most recent pre-pandemic seasons (2014/2015 to 2018/2019) (10). This trend has continued during the 2023–2024 season, for reasons that remain unclear (11).

This study explores the GAS population during Ontario’s iGAS outbreak and identifies potential genetic factors influencing this trend. To do this, we analyzed a collection of 38 invasive (blood) and 117 non-invasive (pharyngeal) *S. pyogenes* isolates from the Greater Toronto Area during the months of January to May in 2023 using whole-genome sequencing (WGS). We compared our isolates both independently and to over 2,000 genomes available in the NCBI database to gain a better understanding of our local epidemiology in the global context. Additionally, we performed genome-wide association analyses and specifically looked for the presence of 63 relevant virulence factor and adhesin genes, antimicrobial resistance (AMR) genes, as well as deletion mutations in a critical two-component system (CovRS), to determine if there were genetic signatures that may explain this increase in iGAS disease.

## RESULTS

### Group A streptococcal isolates are widely distributed, and invasive isolates do not cluster phylogenetically

**Fig 1:**
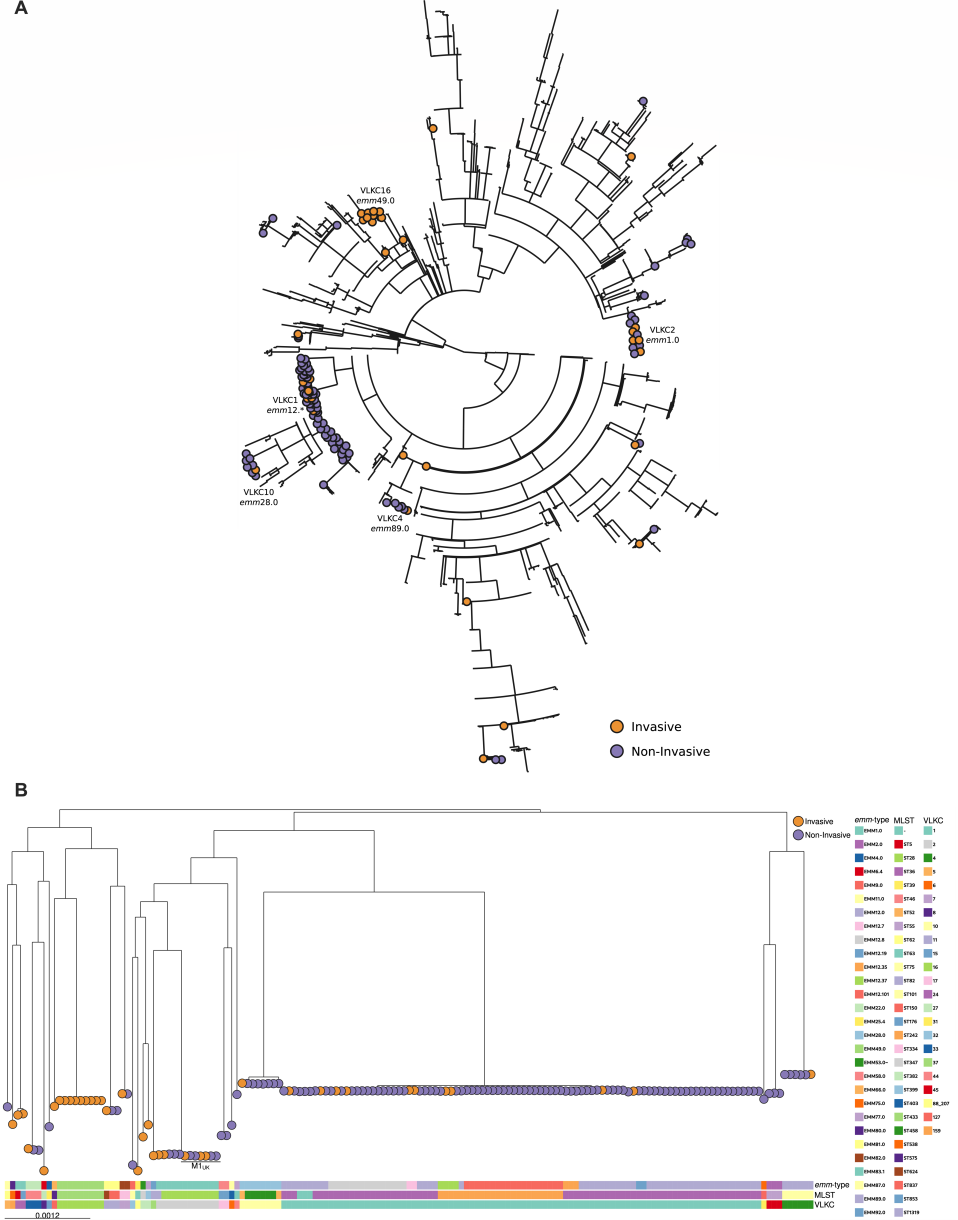
Clinical Group A streptococcal isolates do not cluster based on invasiveness. Whole-genome sequencing analysis was performed on 38 invasive and 117 non-invasive clinical isolates of *Streptococcus pyogenes* collected between January and May 2023. (**A**) A minimum spanning tree of the genomes of the invasive and non-invasive isolates from the current study to over 2,000 published genomes available in the NCBI databank. (**B**) Top, a core genome phylogeny tree of only the isolates in this study. Bottom, three different typing schemes for Group A Streptococcus, *emm*-type, multilocus sequence typing (MLST), and variable length k-mer clustering (VLKC). Each circle represents a clinical isolate and each color indicates whether the isolate comes from an invasive (orange) or non-invasive (purple) sample.

To investigate if there was a genomic explanation for the increase in iGAS cases in Ontario during the early months of 2023, we performed WGS on 38 invasive (blood) and 117 non-invasive (pharyngeal) isolates of *S. pyogenes* collected from a clinical microbiology laboratory that provides service to numerous hospitals in the Greater Toronto Area. We compared our isolates using variable length k-mer clustering (VLKC) to a previously published (12), globally representative, collection of 2,000 GAS genomes and visualized these results using a minimum spanning tree (Fig. 1A). VLKC (as implemented in the tool PopPUNK) involves comparing and grouping genomes based on the relative numbers of shared k-mers across a range of different k-mer sizes (13). This analysis demonstrated that our invasive and non-invasive isolates were broadly distributed across previously sampled GAS diversity with 18.1% (*n* = 28) closely related to only one or two of our other samples. However, 59% of our samples formed a single cluster (VLKC1/*emm*12.*, *n* = 92) with the remaining 22.6% found within four clusters of >3 our genomes (VLKC2/*emm*1.0, *n* = 12; VLKC16/*emm*49.0, *n* = 9; VLKC10/*emm*28.0, *n* = 8; and VLKC4/*emm*89.0, *n* = 6). Except for the entirely invasive VLKC16/*emm*49.0 cluster and 41.7% of the VLKC2/*emm*1.0 cluster, invasive isolates were either singletons or formed a minority of samples within a cluster (10.9–16.6%, respectively).

Next, we wanted to evaluate whether invasiveness formed specific phylogenetic lineages within these clusters. We generated a maximum-likelihood core-genome (1,391 genes) phylogeny tree of our clinical samples. This showed no clear fine-grained phylogenetic structure further explaining invasiveness (Fig. 1B). Similarly, a sub-analysis of the 1,517 core genes of the 92 VLKC1/*emm*12.* samples (Fig. S1) found no clear invasive phylogenetic lineages within this cluster.

### Limited evidence of genes or genomic elements strongly associated with invasiveness

We performed two genome-wide association studies (GWAS) to identify any previously unknown genes, gene clusters, or genomic elements associated with invasiveness (correcting for population structure and multiple comparisons). A single hypothetical gene (group_2020) was significantly associated with invasiveness (*P* < 8.77E−05). This gene was exclusively present in four invasive genomes distributed across three phylogenetically distinct clusters (VLKC1/*emm*12.7, VLKC8/*emm*6.4, and VLKC159/*emm*80.0). BLASTX against NCBI’s non-redundant protein database showed this gene to be of unknown function but associated with several *S. pyogenes* phage genomes. Two sets of overlapping co-located genes (group_148-664-1097 and group 664-1097-1161) were also identified as significantly associated with invasiveness (*P* < 6.46E−05) and were identified in 19 and 7 phylogenetically diverse (nine distinct VLKCs) invasive genomes, respectively. However, these two genes were also associated with 106 and 91 non-invasive isolates, respectively, meaning it is unlikely that these gene clusters have strong penetrance. The genes present in both clusters (group_664 and group_1097) were identified using BLASTX as a YfhO membrane protein and an IS30-family transposase.

### Superantigen and DNase gene abundances differ between invasive and non-invasive isolates

**Fig 2:**
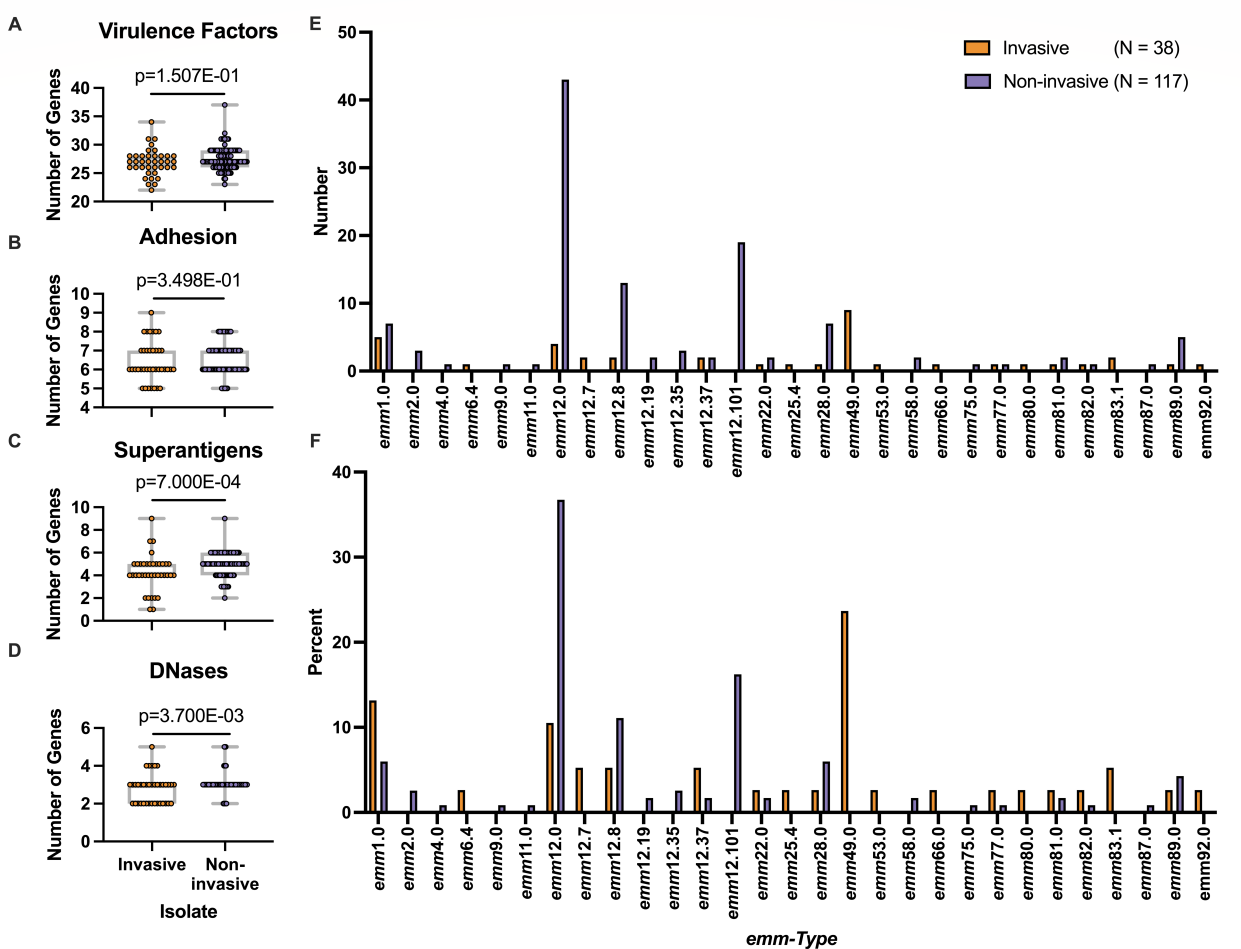
Virulence factor and *emm-*type distribution in invasive and non-invasive clinical isolates of *Streptococcus pyogenes*. The genomes of our clinical isolates were assessed for the presence or absence of numerous relevant virulence factors, adhesins. and their *emm-*type. The gene was considered present if, compared to a reference gene, there was ≥80% sequence match found in the isolate. Comparison was made by adding up all the genes associated with virulence factors (**A**) adhesins (**B**) and subsets of virulence factors including superantigens (**C**) and DNases (**D**). Each circle represents an invasive (orange) or non-invasive (purple) clinical isolate. The box contains all isolates that are between the 25th and the 75th percentiles; and the whiskers extend out to the minimum and maximum values. Mann-Whitney non-parametric comparison was made between the two groups and *P* values are displayed. the *emm*-type distribution of the isolates is shown by comparing the total number (**E**) or percent total (**F**) of each subgroup. Orange bars represent the invasive isolates, and purple bars represent the non-invasive isolates.

Next, we assessed if there were differences in the abundance of known virulence factors or adhesins between our invasive and non-invasive isolates. Virulence factors were subcategorized into distinct functional families (superantigens, DNases, adhesins, or total virulence factors) and assessed for presence/absence in our isolates (by having a sequence with ≥80% identity present compared to a reference gene sequence in their genome) (Table 1). We then compared subcategories of each individual gene between invasive and non-invasive isolates to determine if there was a difference between the two groups. Interestingly, there was no difference in the number of overall virulence factors (Fig. 2A) or adhesins (Fig. 2B) between the two groups. We did find that non-invasive isolates encoded more superantigen (*P* = 7.00E−04) and DNase (*P* = 3.70E−03) genes per genome than did invasive isolates (Fig. 2C and D, respectively). At the individual gene level, we noted several genes that were statistically more common in invasive isolates including the M-like proteins (*enn*, *P* = 4.00E−04; *mrp*, *P* = 1.00E−03), the superantigen *speA* (*P* = 2.10E−03), another virulence factor, *ideS/Mac* (*P* < 1.00E−04), and fibronectin-binding proteins (*fbaA*, *P* = 2.00E−04; *fbaB*, *P* = 3.22E−02). Genes that were more common in non-invasive isolates included the superantigens *speC* (*P* < 1.00E−04), *ssa* (*P* = 5.00E−04) and *smez* (*P* < 1.00E−04), the DNase *spd1* (*P* < 1.00E−04), hyaluronidase *hylp* (*P* = 1.77E−02), additional virulence factors *grab* (*P* = 5.00E−04) and *endoS* (*P* < 1.00E−04), and fibronectin-binding proteins *sfbi/prtf1* (*P* < 1.00E−04), *prtf2* (*P* = 2.66E−02), and *sfbx* (*P* = 1.40E−03) (Table 1).

**TABLE 1:** Virulence factors and adhesins in GAS clinical isolates

Category	Subcategory	Gene	Invasive (*N* = 38)		Non-invasive (*N* = 117)		*P* value
			Number	Percent	Number	Percent	
Virulence factors	M and M-like proteins	*emm*	38	100.00%	117	100.00%	1.000E0
		*enn*	16	42.11%	16	13.68%	4.000E−04
		*mrp*	20	52.63%	27	23.08%	1.000E−03
	Capsule	*hasA*	35	92.11%	109	93.16%	7.314E−01
		*hasB*	36	94.74%	109	93.16%	1.000E0
		*hasC*	36	94.74%	109	93.16%	1.000E0
	Superantigens	*speA*	13	34.21%	13	11.11%	2.100E−03
		*speC*	15	39.47%	92	78.63%	<1.000E−04
		*speG*	38	100.00%	115	98.29%	1.000E0
		*speH*	21	55.26%	82	70.09%	1.142E−01
		*speI*	20	52.63%	80	68.38%	8.340E−02
		*speJ*	8	21.05%	15	12.82%	2.918E−01
		*speK*	5	13.16%	10	8.55%	5.269E−01
		*speL*	2	5.26%	3	2.56%	5.967E−01
		*speM*	5	13.16%	6	5.13%	1.391E−01
		*speQ*	4	10.53%	8	6.84%	4.897E−01
		*speR*	1	2.63%	5	4.27%	1.000E0
		*ssa*	2	5.26%	38	32.48%	5.000E−04
		*smez*	27	71.05%	112	95.73%	<1.000E−04
	DNases	*spnA*	38	100.00%	117	100.00%	1.000E0
		*spdB/mf1*	38	100.00%	117	100.00%	1.000E0
		*sda1*	1	2.63%	0	0.00%	2.452E−01
		*sda2*	0	0.00%	0	0.00%	1.000E0
		*spd1/mf2*	15	39.47%	92	78.63%	<1.000E−04
		*spd3/mf3*	12	31.58%	22	18.80%	1.159E−01
		*spd4/mf4*	0	0.00%	0	0.00%	1.000E0
		*sdn*	2	5.26%	9	7.69%	1.000E0
	Leukocidins and associated genes	*sagA*	38	100.00%	117	100.00%	1.000E0
		*slo*	38	100.00%	117	100.00%	1.000E0
		*nga*	38	100.00%	117	100.00%	1.000E0
	Hyaluronidases	*hlyA*	38	100.00%	116	99.15%	1.000E0
		*hylP*	4	10.53%	36	30.77%	1.770E−02
	Other proteases and virulence factors	*endoS*	28	73.68%	117	100.00%	<1.000E−04
		*scpA*	34	89.47%	100	85.47%	7.852E−01
		*scpC*	38	100.00%	117	100.00%	1.000E0
		*sodA*	38	100.00%	117	100.00%	1.000E0
		*cppA*	38	100.00%	117	100.00%	1.000E0
		*grab*	27	71.05%	110	94.02%	5.000E−04
		*ideS/Mac*	18	47.37%	15	12.82%	<1.000E−04
		*sic*	5	13.16%	7	5.98%	1.685E−01
		*speB*	38	100.00%	117	100.00%	1.000E0
		*s5na*	38	100.00%	117	100.00%	1.000E0
		*cfa*	37	97.37%	117	100.00%	2.452E−01
		*htrA/degP*	38	100.00%	117	100.00%	1.000E0
		*ska*	38	100.00%	117	100.00%	1.000E0
		*slaA*	3	7.89%	3	2.56%	1.579E−01
		*spyA*	38	100.00%	117	100.00%	1.000E0
Adherence and other binding proteins	Fibronectin-binding proteins	*fbaA*	24	63.16%	34	29.06%	2.000E−04
		*fbaB*	4	10.53%	2	1.71%	3.220E−02
		*fbp54*	38	100.00%	117	100.00%	1.000E0
		*sfbI/prtF1*	9	23.68%	72	61.54%	<1.000E−04
		*sfbII/sof*	12	31.58%	34	29.06%	8.387E−01
		*prtF2*	26	68.42%	101	86.32%	2.660E−02
		*sfbx*	28	73.68%	110	94.02%	1.400E−03
	Collagen-binding proteins	*cpa*	1	2.63%	0	0.00%	2.452E−01
	Laminin-binding proteins	*lmb*	38	100.00%	117	100.00%	1.000E0
	Plasmin receptor	*plr/gapA*	38	100.00%	117	100.00%	1.000E0
	Collagen-like proteins	*sclA*	20	52.63%	54	46.15%	5.758E−01
		*sclB*	6	15.79%	7	5.98%	8.690E−02

### Prevalence of *emm-*types is different based on invasiveness

The *emm*-type distribution in our clinical isolates was also assessed (Fig. 2E and F; Table S1). We found that our non-invasive isolates were predominantly emm12.*-type (70.09%) and only 5.98% were *emm1*, whereas our invasives were mostly emm12.*, *emm49.0*, and *emm1-*types (26.32%, 23.68%, and 13.16%, respectively). Given the global importance of *emm1* GAS isolates and their ability to cause severe disease, as well as the emergence of the hypervirulent M1_UK_ strain, we sought to determine how many of our isolates had the 27 M1_UK_-specific single nucleotide polymorphisms (SNPs) (7). After analyzing our *emm1* sequences, we found that 58.33% (7 out of 12) of our isolates had all 27 SNPs; however, this finding had no relatedness to invasiveness as our samples had an almost equal distribution of invasive (3 out of 5; M1_UK_/*emm1* total; 60%) to non-invasive (4 out of 7; M1_UK_/*emm1* total; 57.14%) isolates.

### AMR genes are rare in both blood and pharyngeal isolates

The prevalence of AMR genes in all our isolate genomes was relatively low overall (Table S4), and there were no significant differences between our invasive and non-invasive isolates in any of the 11 AMR genes assessed (Table 2). Only 9.68% (15 out of 155) of all organisms were carrying at least one gene, with 15.79% (6 out of 38) of invasive isolates and 7.69% (9 out of 117) of non-invasive isolates. The most common AMR gene by far was *tetM* at 7.74% (12 out of 155) and 8.39% (13 out of 155) of isolates carried at least one *tet* gene (*tetM* or *tetO*). The second most common genes were *aph(3′)-IIIa*, *sat4*, and *ermB,* all with 3.23% (5 out of 155). No AMR gene was found alone, with a range of two to five genes being present in all resistant organisms.

**TABLE 2:** Antimicrobial resistance genes present in GAS clinical isolates

Antibiotic class	Gene	Invasive (*N* = 38)		Non-invasive (*N* = 117)		*P* value
		Number	Percent	Number	Percent	
Aminoglycoside	A*NT (6)-Ia*	2	5.26%	0	0.00%	5.890E−02
	APH(3′)-IIIa	2	5.26%	3	2.56%	5.967E−01
Trimethoprim	dfrG	1	2.63%	0	0.00%	2.452E−01
Macrolide	mefA	2	5.26%	1	0.85%	1.490E−01
Macrolide/streptogramin	msrD	2	5.26%	1	0.85%	1.49E−01
Macrolide/lincosamine/streptogramin	ermA	1	2.63%	3	2.56%	1.000E0
	ermB	0	0.00%	5	4.27%	3.349E−01
	ermT	2	5.26%	0	0.00%	5.890E−02
Streptothricin	Sat4	2	5.26%	3	2.56%	5.967E−01
Tetracyclines	tetM	5	13.16%	7	5.98%	1.685E−01
	tetO	0	0.00%	1	0.85%	1.000E0

### Phenotypic antimicrobial susceptibility patterns match AMR genes

All tested invasive GAS isolates were sensitive to penicillin and vancomycin (Table S4). The highest rate of drug resistance was erythromycin at 13.51% (5 out of 37), with 8.11% (3 out of 37) of isolates bring resistant to clindamycin. All clindamycin-resistant organisms were also resistant to erythromycin. Phenotypic AST results synced up with our genetic screen, with all erythromycin-only resistant organisms having one of *mefA* or *msrD*, and all double-resistant organisms having one *erm* gene (*ermA* or *ermT*). Three organisms tested intermediate to clindamycin, but these organisms had no detectable AMR genes.

### Non-invasive isolates have larger zones of hydrolysis and hemolysis

**Fig 3:**
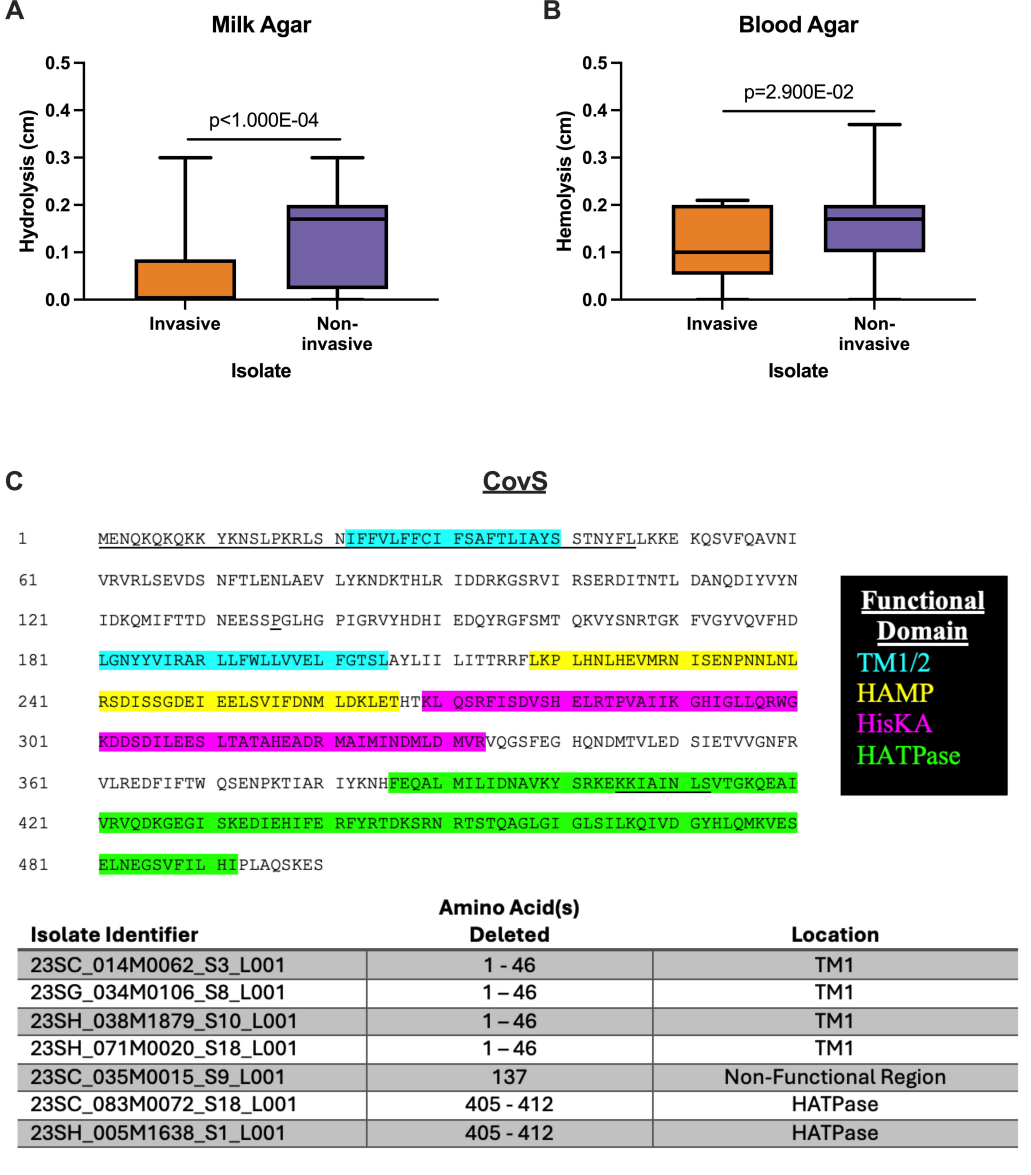
Phenotypic differences and deletion mutations in the CovS protein in clinical isolates. *Streptococcus pyogenes* clinical isolates were cultured for 24 h at 37°C in ambient air and zones of clearance were measured on (**A**) milk agar (hydrolysis) and (**B**) blood agar (hemolysis). The zone of clearance for each organism was determined by averaging triplicate measurements. Graphs represent box and violin plots with the box representing the middle quartiles, the middle horizontal line indicating the median, and the whiskers extending to the minimum and maximum values measured. Stated *P* values were determined by Mann-Whitney test. (**C**) Translated CovS protein sequences were assessed for deletion mutations in all GAS clinical isolates with only seven invasive isolates being found to have these mutations. (Top) The CovS protein sequence with the location of each deletion mutation underlined. The legend (right) outlines the functional domains in the CovS protein (Transmembrane Helix, TM, Blue; present in Histidine kinases, Adenyl cyclases, Methyl-accepting proteins and Phosphatases, HAMP, yellow; histidine kinase domain [phosphoacceptor], HisKA, magenta; histidine-kinase like ATPase, HATPase, green). (Bottom) A table outlining the invasive isolates that had CovS mutations, the length of the deletion, and the location of the deletion.

To measure SpeB protease activity (14) and streptolysin S (SLS) activity (15), we plated each of our clinical isolates on two Columbia-based culture media, one containing 3% skim milk (milk agar) and the other containing 5% sheep’s blood (blood agar), respectively. After 24 h of incubation at 37°C in ambient air conditions, zones of clearance measurements (milk agar, casein hydrolysis, blood agar, and hemolysis) in triplicate were recorded from each isolate on each agar and graphed (Fig. 3A and B). We observed in both assays that our pharyngeal isolates had statistically larger zones of clearance (milk agar, *P* < 1.00E−04; blood agar, *P* = 2.90E−02). The percentage of invasive isolates that had no measurable SpeB production (a casein hydrolysis zone of zero) was 63.63% (21 out of 33), whereas 24.51% (25 out of 102) of our pharyngeal isolates displayed the same phenotype. For SLS activity, 23.68% (9 out of 38) of our invasive isolates showed no beta-hemolysis compared with 5.36% (6 out of 112) of our pharyngeal isolates (Table S2).

### CovS deletions are present only in GAS-invasive isolates

Given that the two-component system CovRS influences transcription of 15% of all chromosomal genes (16), and mutation in either component can result in a more virulent GAS phenotype (17–19), we sought to assess whether these mutations were present in our samples. We compared the translated *covR* and *covS* sequences in all our isolates to see if deletion mutations had occurred. Surprisingly, there were no deletions seen in any *covR* sequences (data not shown). In *covS*, we observed deletions in seven of our invasive isolates and none of the non-invasive isolates (Fig. 3C). These deletions ranged from 1 to 46 amino acids in length and almost always (six out of seven; 85.71%) occurred in an important functional domain (transmembrane helix or the histidine-kinase like ATPase).

## DISCUSSION

This study describes the genomic characterization of both invasive (blood) and non-invasive (pharyngeal) clinical GAS isolates from Toronto, Canada in 2023, during a surge of invasive cases. We performed WGS on these strains and investigated their relatedness in terms of VLKC, *emm-*typing, and core genome phylogenies. Additionally, we performed both GWAS analyses and characterized the distribution of known virulence genes (including deletions in the CovRS two-component system) to identify genomic signatures associated with invasiveness.

A difference between *emm*-types was observed between invasive and non-invasive cohorts with *emm12* dominating pharyngeal isolates (70.09%) and *emm12* (26.32%), *emm49* (23.68%) and *emm1* (13.16%) most common among our invasive isolates (Fig. 2E and F; Table S1). This result is consistent with surveillance data from local public health reporting during the same timespan (10). Surprisingly, in both data sets, there was almost a complete lack of the *emm*74 lineage of *S. pyogenes* that was previously seen in Ontario and was an epidemic subgroup of iGAS in Canada during 2016 and 2017 (20). The most recent Canada-wide surveillance data from 2022 (21) outlined *emm*49 (16.9%) as the predominant *emm-*type, with *emm74* making up ~9% of all isolates, *emm*1 making up less than 5% of iGAS isolates, and *emm*12 making up just under 6%. Data from British Columbia in 2023 showed a somewhat similar *emm*-type distribution to ours with *emm*12, *emm*49, and *emm*1 as predominant subtypes (19%, 15%, and 10% of iGAS, respectively); however, *emm*74 was still circulating and made up around 11%. These data taken together indicate that there are similarities in major circulating *emm*-types nationally that cause iGAS; however, epidemic strains that are no longer circulating in one part of the country may still be present in others, and this can shift over a relatively short period of time. It is also important to note that there is a lack of nasopharyngeal surveillance of GAS in Canada and worldwide. Given the relative genomic diversity observed in this small study, broader community and hospital-based pharyngeal surveillance studies may allow us to better understand the dynamics and invasiveness of GAS.

Another interesting finding was that *emm49* strains from our study were only seen in invasive (blood) isolates. This is of note because this GAS subtype falls within the *emm* pattern group E, which has previously been observed in equal proportions in both throat (pharyngeal) and skin (impetigo) clinical specimens (22), which led us to expect to find these isolates in our pharyngeal clinical samples. Recent studies, however, have found the majority of *emm49* clinical isolates to be recovered from invasive sites (23) and when they are causing non-invasive disease, it is far more likely to be on the skin (impetigo) (24). Additionally, de Crumbrugghe et al. showed that when *emm49* isolates do exist in the nasopharynx, they are far more likely to exist in an asymptomatic carriage state (25). This explains the lack of *emm49* strains in our non-invasive pharyngeal isolates; clinical specimens from the nasopharynx are generally only collected from symptomatic patients due to the non-sterile nature of the location. Future studies could assess what factors allow *emm49* isolates to cause such a dynamic range of clinical symptoms from these diverse anatomical locations.

The hypervirulent M1_UK_ clone, complete with all 27 subset-defining SNPs (7), also made up a majority of the *emm*1 isolates in our study (7 out of 12; 58.33%). This was not unexpected given that it had already been found in Canada (9) and it has been shown to outcompete other *emm1*-subtypes in other settings (8). However, it was interesting that an almost equal split between invasive (three out of five; 60%) and non-invasive (four out of seven; 57.14%) M1_UK_ clones was observed in our data set. It is known that the M1_UK_ clone can cause both pharyngeal (scarlet fever) and invasive disease phenotypes (7), but the prevalence at which it causes either disease manifestation is unknown. Our study shows that genomic analysis alone cannot consistently differentiate invasive from non-invasive M1_UK_ clones. Future studies should examine phenotypic and host factors to elucidate what determines the tropism of this emerging variant.

Our study found no difference in the overall presence of all 63 analyzed virulence factors or adhesins (Table S3) between our two groups of isolates (Fig. 2A and B). Interestingly, we noted that our pharyngeal isolates had a higher number of superantigen genes compared to our invasive isolates (Fig. 2C). Although superantigens are best understood in the context of their contribution to severe diseases like toxic shock syndrome (26), there is research that supports the role of these toxins in nasopharyngeal colonization in a murine model of pharyngitis (27, 28), and thus it is conceivable that this could also be true in the context of human pharyngeal infection/colonization. The composition of superantigen genes differed between the groups as invasive isolates were found to contain the *speA* gene more often, whereas pharyngeal isolates were more prevalent in *speC*, *ssa*, and *smez*. Given that the acquisition of *speA* has previously been shown to drive severe GAS disease (29, 30) these results are not unexpected. Overall, there was no combination of superantigens that perfectly predicted invasiveness. Much like what has been shown using a non-human primate model of pharyngitis (31), it is likely that regulation of these toxins is important to their contribution to colonization/infection and future research should focus on determining if there is a difference in superantigen expression between invasive and non-invasive GAS clinical isolates.

We also observed that our pharyngeal isolates harbored more DNase genes compared to our invasive isolates (Fig. 2D), of which *spd1* was statistically more common in these isolates (Table 1). Streptococcal DNases have been best described for their ability to evade the host immune system, iconically by degradation of the chromatin found in neutrophil extracellular traps (NETs) (32, 33), which would lend their activity to being most important during invasive infection (34, 35). However, there is an evolving literature on their role in nutrient-savaging, niche competition, and communication (36). Interestingly, in numerous publications *spd1* was found to be crucial for nasopharyngeal colonization (37) and increased shedding (38) in murine infection models. These findings, along with our data, support the idea that DNases are not only important during invasive infections, but may play a critical role in upper respiratory tract niche adaptation and bacterial spreading. Future research would benefit from looking further at this interesting result.

Other individual factors were also found to have differential prevalence based on invasiveness. The fibronectin-binding protein *fbaA* and *fbaB* were found to be more prevalent in our invasive isolates, whereas *prtF1*, *prtF2*, and *sfbx* were more prevalent in pharyngeal isolates (Table 1). There has been little to no clinical data associating any of these factors with invasive disease; however, one study (39) looked at the *fbaA* gene prevalence in pharyngeal versus skin-infecting (impetigo) strains and found a higher prevalence in the impetigo strains, indicating that the invasive isolates in our study may be more likely to come from skin infections or skin colonizing strains of GAS than from those that inhabit the nasopharynx. A second study found that GAS strains that expressed *prtF1* were less virulent in a murine intraperitoneal infection model (40), which may indicate why our invasive isolates had a lower prevalence of this gene. Finally, *ideS*/*Mac* was more prevalent in our invasive isolates, whereas *grab* and *endoS* were more prevalent in our pharyngeal isolates, and although the functions of these genes have been well described (1, 2, 41), their association with clinical disease has been understudied and thus their overall contribution to invasiveness is difficult to interpret.

The phenotypic resistance patterns observed in our invasive clinical isolates are similar to those observed in other recently published Canadian literature around the same time as our study (21). The one major difference between our study and this report was the percentage of clindamycin resistance which was found to be relatively stable between 2018 and 2022 at 2.9–4.8% by Golden et al., whereas our study saw 8.1% (3 out of 37) resistance. This could indicate the start of an increase in the prevalence of clindamycin resistance in Canada or could be due to a bias of a smaller sample size and future research should keep an eye on this trend.

The tracking of GAS AMR genes in Canada is far less common than the tracking of phenotypic susceptibility profiles. When AMR genes are tracked, it is usually done in an *emm*-subtype specific outbreak scenario (42), or for local surveillance (43). Additionally, these older studies tend to use a more targeted method such as PCR, which although valid, may miss genes that you would detect using a more unbiased screening tool like WGS. A more recent study using a similar genomics approach to ours assessed AMR genes from non-invasive throat and skin isolates from 2016 to 2017 and 2022 to 2023 in the United Kingdom (24). In their 2022–2023 pharyngeal isolates, they found an overall higher rate of *tetM* (13.88%; 29 out of 209), *ermA* (4.31%; 9 out of 209), and *mefA* (1.91%; 4 out of 209), whereas our isolates had more *ermB* (4.27%; 5 out of 117). Starting to include a genetic AMR component to the regularly released iGAS reports by the Public Health Agency of Canada as well as the provincial and territorial public health laboratories would allow for an additional level of antibiotic resistance surveillance in the GAS isolates of this country. Starting a national GAS pharyngeal and skin screening program would also provide invaluable information about the GAS strains circulating in this country which go undetected in the hospital systems.

The interplay of host factors, biases in sampling, and the likely existence of multiple lineage-specific genetic factors driving invasiveness greatly reduced the utility of GWAS for a data set of this size. However, these analyses did identify two weakly penetrative sets of co-located genes and a rare hypothetical gene with a significant and highly penetrative invasive disease association. This latter gene was found in several unrelated genomes and was linked to GAS phage sequences in NCBI. Therefore, it is highly likely this gene has the potential to undergo lateral gene transfer. Given the potential public health significance of a mobile gene associated with invasive disease, future work is needed to characterize the functional role of this gene.

Our study had several limitations, including that the isolates were from only one geographical region and that there was no associated patient data. Although our invasive strains all came from blood cultures, which is indicative of iGAS disease, we do not know if our pharyngeal isolates came from individuals with pharyngitis or included individuals with other GAS disease manifestations. Second, considering other groups that have associated the M1_UK_ strain and its overproduction of the superantigen SpeA to causing scarlet fever (7), future studies would benefit from combining richer clinical and WGS data to determine if there are any genetic correlates to specific disease manifestations. It is also well known that host factors may contribute to individuals being more likely to develop severe disease (44). Access to information on allelic carriage of infected individuals would also help further understanding of this important association.

The presence of a virulence factor or adhesin gene in the genome does not provide information on whether that gene is expressed. Our study measured zones of casein hydrolysis and beta-hemolysis in ambient air conditions as proxies for protease SpeB (14) and SLS activity (15), respectively. We found that pharyngeal isolates had larger zones of clearance in both assays (Fig. 3A and B), suggesting that SpeB and SLS are produced in higher abundances in these isolates. Additionally, 63.63% of our invasive isolates and 24.51% of our pharyngeal isolates demonstrated no SpeB production at all (zone of hydrolysis on milk agar = 0.00 cm). These findings differ from what Feng et al. found, where 37.5% of their invasive and 12% of their pharyngeal isolates showed no SpeB production (45). Olsen et al. also noted that in analyzing over 6,700 GAS isolates from human infections, 84.3% had a wild-type SpeB protease phenotype (46). There could be numerous reasons for these discrepancies including variations in the assays used to measure SpeB protease activity, the year in which GAS isolates were collected as well as the *emm-*type distribution of clinical isolates tested. Future studies should use a more standardized approach to assessing virulence factor production and may benefit from looking at transcriptomics to further explore these and other important virulence factors in clinical GAS strains (47).

We also examined deletion mutations in the *covS* gene, which is known to impact the expression of numerous key virulence genes, including SpeB (18, 19). Indeed, we only saw *covS* deletions in our invasive isolates (Fig. 3C), and these isolates showed no casein hydrolysis supporting their lack of SpeB production (Table S2). Interestingly, *covS* was not universally affected in all isolates, as only 18.42% had such deletions. Other groups have also seen disproportionate rates of *covS* mutations in their invasive versus pharyngeal clinical isolates of GAS (23). These data further support the notion that deletions in this gene are associated with an increase in invasive disease.

In summary, our study identified significant differences in virulence factor genes, *emm*-types, and mutations in a two-component system between invasive and non-invasive clinical GAS isolates, highlighting the importance of genomic surveillance in managing iGAS infections. The continued investigation of GAS using WGS will not only allow us to track epidemics and emerging pathogenic strains of importance, but combined with looking at specific virulence factors will allow us to determine the appropriate targets for future therapies and vaccination strategies which would help curb the burden caused by the globally important pathogen.

## MATERIALS AND METHODS

### Clinical isolate collection and storage

Group A streptococcal strains were isolated from pharyngeal (non-invasive) or blood (invasive) clinical specimens collected as part of routine clinical care between 7 January and 31 May 2023 (Fig. S2). Clinical specimens were received by the laboratory, processed, and incubated on routine media (BBL Columbia Agar with 5% sheep blood [BAP] or BBL Chocolate II Agar [BD Diagnostics]) for up to 48 h at 37°C + 5% CO_2_ or ambient air conditions. Bacterial identification was determined using MALDI-ToF (Bruker Diagnostics). Upon confirmation of bacterial identification, isolates were frozen in tryptic soy broth with 20% glycerol (vol/vol) and stored at −70°C or used directly for antimicrobial susceptibility testing (see below). Full isolate information can be found in Table S2.

### DNA extraction

Isolates were thawed and plated on BAP and grown at 37°C + 5% CO_2_ for 24–48 h and assessed for purity. Several bacterial colonies were resuspended in a sterile tube containing 200 µL TE buffer (MilliporeSigma). The suspension was pre-treated with 1 µL of Ready-Lyse Lysozyme Solution (LGC Biosearch Technologies) and incubated for 30 min at 37°C. The lysed sample was then extracted on the NucliSENS easyMag (BioMérieux). The genomic DNA concentration of extracts was determined using the Qubit 4 Fluorometer with the Qubit 1X dsDNA HS kit (Invitrogen Life Technologies).

### Library preparation and WGS

Libraries were prepared using the Illumina DNA Prep kit (Illumina) with IDT for Illumina UD Indexes (Illumina) according to the manufacturer’s instructions. Individual libraries were quantified using the Qubit 4 Fluorometer with the Qubit 1X dsDNA HS kit and normalized to equimolar concentrations. Pooled libraries were analyzed on the TapeStation 4150 using D1000 ScreenTape (Agilent) and sequenced on the MiniSeq using a 300-cycle HighOutput Reagent kit (Illumina) in paired-end mode (2 × 149 bp).

### Genomic assembly and annotation

Clinical isolates (*n* = 155) were assembled using Bactopia v3.0.0 (48) workflow with default settings. In brief, this involves QC with fastp v0.23.4 (49) followed by assembly using shovill v1.1.0 (https://github.com/tseemann/shovill) and annotation using prokka v1.14.6 (50) and amrfinderplus v3.11.18 (with database v2023-08-08.2) (51). Via Bactopia subworkflows/tools the following analyses were also performed: multilocus sequence typing (MLST) using mlst v2.23.0 (https://github.com/tseemann/mlst) against the 2.23.0-20230907 database and Enright et al. scheme (52), *emm*-types inferred using emmtyper v0.2.0 (https://github.com/MDU-PHL/emmtyper).

### Genome clustering and phylogenetics

Recombination masked core genome maximum-likelihood phylogenies were inferred for all isolates and just *emm*12 isolates using panaroo v1.3.4 (53), clonalframeml v1.12 (54), and iqtree v2.2.2.7 (55). All clinical isolates were then clustered into VLKCs using a curated reference set of 2,085 assembled *S. pyogenes* genomes with poppunk v2.6.2 and the spyogenes v1.0.0 database (13). A minimum spanning tree was then inferred from cluster assignments via poppunk. Both the core genome phylogeny of clinical samples and the minimum spanning tree of clinical and representative contextual genomes were visualized using microreact (56).

### Clinical isolate exclusion criteria

GAS strains that were isolated from the same anatomical site in the same patient within 48 h and were found to be genetically similar by WGS were considered duplicates and removed from the analysis (*n* = 2).

### Invasiveness GWAS

Tested for the significant association of individual genes with invasiveness was performed using pyseer v1.3.11 (57, 58) with the panaroo-inferred gene presence and absence table and a kinship matrix calculated using patristic distances in the core genome phylogeny. A random effect linear mixed model was used to correct for population structure, and the significance threshold was calculated based on 1,373 filtered patterns tested (*P* value threshold of 8.77E−05). Significant genes were then compared against collated metadata and further annotated using NCBI’s BLASTX web portal (using BLAST+ 2.16.0 and the 2024-08-19 non-redundant protein database). This process was then repeated for co-located groups of genes using panaroo-inferred structure presence and absence data (1,474 filtered patterns and a *P* value threshold of 6.46E−05).

### GAS virulence and adhesin genes of interest selection and comparison

A customized set of virulence and adhesion factors (Table S3) were selected based on their presence in the Virulence Factor Database (VFDB) (59), RefSeq (60), and in relevant literature searches (1, 2). They were annotated using BLASTN+ v2.15.0 (61) with a minimum identity and query coverage of 80%. Alignments were also generated using minimap2 (62) and gofasta (63) against the MGAS8232 (64) *covR* and *covS* reference sequences. Functional regions in *covS* were determined by NCBI GenBank: AAF00082.2 and relevant publications (19, 33).

### AMR genes and phenotypic antimicrobial susceptibility testing

AMR gene carriage was determined using AMRFinderPlus v3.12.8 (51) with database v2024-07-22.1. Antimicrobial susceptibility testing data were downloaded from the laboratory information system. Phenotypic susceptibility testing was performed using disk diffusion methodology according to Clinical and Laboratory Standards Institute 2022 guidelines and interpretations. Briefly, Colony suspensions were made using normal saline (0.85% NaCl) equivalent to a 0.5 McFarland standard from colonies grown overnight (18–20 h) on a BAP. The suspension was inoculated onto Mueller-Hinton agar with 5% sheep blood (BD Diagnostics) using a culture swab and incubated at 37°C + 5% CO_2_ for 20–24 h with the indicated antibiotic-impregnated disks (penicillin, 10 units; clindamycin, 2 µg; erythromycin, 15 µg; and vancomycin, 30 µg). Zones of inhibition were measured with a ruler and interpreted according to CLSI 2022 guideline values. Results of genotypic and phenotypic results can be found in Table S4.

### Hydrolysis and hemolysis assays

Clinical isolates were subcultured from frozen on a BAP and incubated overnight (18–24 h) at 37°C in ambient air conditions. Multiple individual colonies were picked and platted on Columbia agar with 3% skim milk (skim milk agar, casein hydrolysis, Bio-Media Unlimited Ltd., Toronto, Canada) and BAP (hemolysis) to achieve single-colony resolution and incubated at 37°C in ambient air conditions. At 24 h, plates were removed from the incubator, and the zones of hemolysis (BAP) and casein hydrolysis (skim milk agar) were measured using a standardized ruler. Triplicate measurements were taken for each isolate and averaged. For technical reasons, 33 invasive isolates and 103 pharyngeal isolate measurements were included in the final hydrolysis assay measurements, and 112 pharyngeal isolates were included in the final hemolysis assay measurements (all invasive isolates were also included). The average zone of clearance sizes can be found in Table S2.

### Statistical analysis

Statistical associations between individual genes or groups of genes were evaluated between our invasive and non-invasive isolates. Mann-Whitney non-parametric comparison was done between the two groups for comparison of virulence factor groups (Fig. 2A through D) and Fisher’s exact test was performed for individual genes and *P* values are displayed in Tables 1 and 2. Statistical analyses were performed in either GraphPad Prism (version 10.2.2; Mann-Whitney) or JMP Pro (version 17.2.0; Fisher’s exact).

## Data Availability

Raw sequencing and assembly data are available for all samples in NCBI under BioProject PRJNA1149826. All genomic analysis code and intermediate outputs are available at DOI: 10.5281/zenodo.13346035.

## SUPPLEMENTAL MATERIAL

Supplemental figures spectrum.02141-24-s0001.docx Figs S1 to S3.

Supplemental tables spectrum.02141-24-s0002.xlsx Tables S1 to S4.

ASM does not own the copyrights to Supplemental Material that may be linked to, or accessed through, an article. The authors have granted ASM a non-exclusive, world-wide license to publish the Supplemental Material files. Please contact the corresponding author directly for reuse.
