# Supplementary material for: Comparison of pharyngeal and invasive isolates of Streptococcus pyogenes by whole-genome sequencing in Toronto, Canada
Source: Microbiol Spectr. 2025 Feb 13;13(4):e02141-24. doi: 10.1128/spectrum.02141-24 (PMC11960128; doi:10.1128/spectrum.02141-24)
Supplement: Supplemental figures — Figs S1 to S3. [file spectrum.02141-24-s0001.docx]

**Figure S1**: Distribution of invasive and non-invasive *emm*12* clinical *Streptococcus pyogenes* isolates. Top, core genome phylogeny tree of *emm*12* isolates in this study. Bottom, *emm*12-subtype and Multilocus sequence typing (MLST) colour-coded according to the figure legend. Each circle represents a clinical isolate and each colour indicates whether the isolate come from an invasive (orange) or non-invasive (purple) sample.


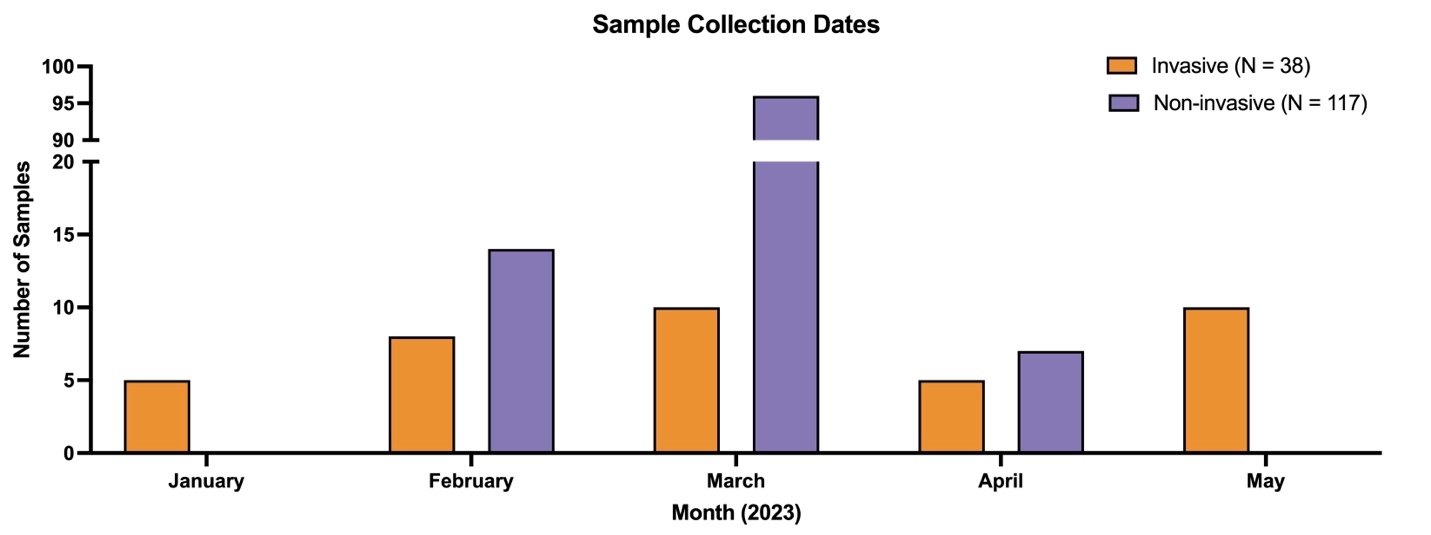


**Figure S2: Number of Group A Streptococcal clinical isolates collected per month in 2023**. Single confirmed colonies of *Streptococcus pyogenes* grown on nutrient rich agar were collected from patient samples and stored at -70 degrees Celsius in tryptic soy broth plus 20% glycerol. Organism identification was confirmed by MALDI-ToF. Non-invasive isolates (purple bars) came from patient throat swabs while invasive isolates (orange bars) came from blood samples.
